# Fibroblast Growth Factor Receptor (FGFR) Signaling in GIST and Soft Tissue Sarcomas

**DOI:** 10.3390/cells10061533

**Published:** 2021-06-17

**Authors:** Andrea Napolitano, Alexandra E. Ostler, Robin L. Jones, Paul H. Huang

**Affiliations:** 1 Sarcoma Unit, The Royal Marsden NHS Foundation Trust, 203 Fulham Road, London SW3 6JJ, UK; andrea.napolitano@rmh.nhs.uk (A.N.); Alex.Ostler@rmh.nhs.uk (A.E.O.); robin.jones4@nhs.net (R.L.J.); 2 Department of Medical Oncology, University Campus Bio-Medico, 00128 Rome, Italy; 3 The Institute of Cancer Research, 237 Fulham Road, London SW3 6JB, UK

**Keywords:** sarcoma, gastrointestinal stromal tumor, fibroblast growth factor receptors, tyrosine kinase inhibitors

## Abstract

Sarcomas are a heterogeneous group of rare malignancies originating from mesenchymal tissues with limited therapeutic options. Recently, alterations in components of the fibroblast growth factor receptor (FGFR) signaling pathway have been identified in a range of different sarcoma subtypes, most notably gastrointestinal stromal tumors, rhabdomyosarcomas, and liposarcomas. These alterations include genetic events such as translocations, mutations, and amplifications as well as transcriptional overexpression. Targeting FGFR has therefore been proposed as a novel potential therapeutic approach, also in light of the clinical activity shown by multi-target tyrosine kinase inhibitors in specific subtypes of sarcomas. Despite promising preclinical evidence, thus far, clinical trials have enrolled very few sarcoma patients and the efficacy of selective FGFR inhibitors appears relatively low. Here, we review the known alterations of the FGFR pathway in sarcoma patients as well as the preclinical and clinical evidence for the use of FGFR inhibitors in these diseases. Finally, we discuss the possible reasons behind the current clinical data and highlight the need for biomarker stratification to select patients more likely to benefit from FGFR targeted therapies.

## 1. Introduction

Sarcomas are a heterogeneous group of malignancies of mesenchymal origin. In the last World Health Organization Classification of Soft Tissue and Bone Tumors [1], more than 100 different histologically and/or molecularly characterized mesenchymal tumors were described, the majority of which are soft-tissue sarcomas (STS). In adults, STS are classified as rare cancers, accounting for about 1% of all malignancies. The annual incidence for each individual STS subtype ranges from ~15 cases to less than 0.1 cases per million persons [2]. Gastrointestinal stromal tumors (GIST) are the most common type of mesenchymal tumor. Among the STS, the most frequent subtypes are leiomyosarcomas (LMS), undifferentiated pleomorphic sarcomas (UPS), and liposarcomas [2]. In the pediatric and young adult population, the incidence of cancer is significantly lower; however STS are relatively more frequent, representing between 7–10% of all malignancies [3]. In this age group, the most common histologies are rhabdomyosarcoma (RMS) and synovial sarcoma [3]. In the last few decades, several advances have been made in our understanding of the molecular events associated with the pathogenesis and progression of different sarcoma subtypes. A complex signaling pathway with crucial implication in these processes depends on the interactions between fibroblast growth factors (FGFs) and their cognate receptors, the fibroblast growth factor receptors (FGFRs) [4,5].

Briefly, FGFs are a large family of structurally related signaling molecules that can be divided into three main groups. The first two groups are: canonical FGFs (FGF 1–10, 16–18, 22), which are secreted proteins capable of binding to heparin and heparan sulphate with high affinity, mostly displaying autocrine and paracrine action; and endocrine FGFs (FGF 15/19, 21, 23), which are also secreted proteins but have a lower affinity to heparin and heparan sulfate and therefore present an endocrine action. Both of these groups mediate their biological responses in a FGFR-dependent manner. The last group comprises intracellular FGFs (FGF 11–14), which are not secreted and have no identified interaction with FGFRs [6].

There are four main FGFRs (FGFR 1–4) with several splice variants, characterized by different cellular expression as well as ligand binding specificity and affinity. FGFRs consist of an extracellular ligand domain, a transmembrane domain, and an intracellular domain with tyrosine kinase activity. Their activation depends on homo- or hetero-dimerization and subsequent trans- and auto-phosphorylation of the kinase domain [6]. Activated FGFRs are coupled to several intracellular signaling pathways including the rat sarcoma (RAS)–mitogen-activated protein kinase (MAPK) and phosphoinositide 3-kinase (PI3K)–protein kinase B (PKB) pathways [6]. Among the signal transducing adaptor proteins, fibroblast growth factor receptor substrate 2 (FRS 2) represents a pivotal linking protein [7]. A fifth receptor (FGFR 5/FGFRL 1) lacking the tyrosine kinase domain has recently been identified, and it could act as a decoy receptor by negatively regulating FGFR signaling, or more likely as a non-tyrosine kinase signaling molecule [6].

**Figure 1:**
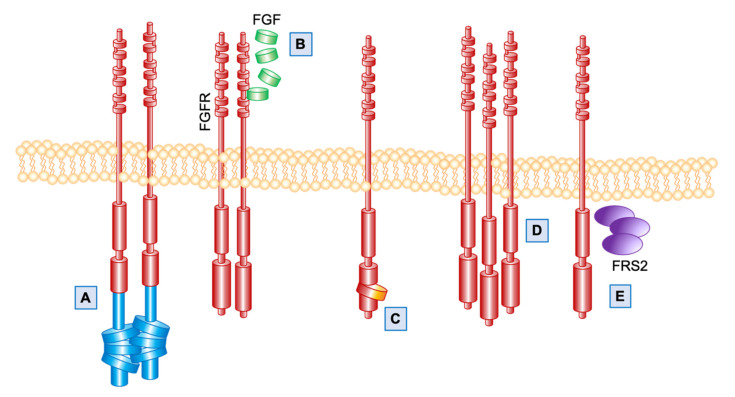
FGFR pathway alterations described in STS. (**A**) FGFR gene fusions; (**B**) overexpression of FGF ligands; (**C**) FGFR activating mutations; (**D**) FGFR overexpression (amplification or transcriptional modulation); (**E**) amplification of the FGFR adaptor protein FRS2.

Overall, it is estimated that about 4% of all sarcoma patients have a targetable alteration in FGFRs [8] (Table 1). Here, we will review the roles of FGFs/FGFRs signaling in the pathogenesis and natural history of selected subtypes of STS, and the potential clinical applications of FGFR inhibitors in these diseases (Figure 1).

## 2. The Roles of FGF/FGFR Pathway in GIST and STS

### 2.1. Gastrointestinal Stromal Tumors

GIST are malignancies generally arising in the gastrointestinal tract from the transformation of the interstitial cells of Cajal, physiologically serving as electrical pacemakers of the gastrointestinal tract. In more than 90% of the cases, driver activating mutations can be found in the tyrosine kinases c-KIT or platelet-derived growth factor receptor (PDGFR)-α. Tyrosine kinase inhibitors (TKIs) such as imatinib and sunitinib represent standard treatments in advanced GIST cases harboring these mutations. More rarely, a proportion of GIST patients do not carry mutations in either kinase and might present mutations in other driver genes including genes belonging to the RAS pathway and *SDHB*, which encodes for a subunit of the enzyme succinate dehydrogenase [19].

In the subset of GIST without any of these alterations (so called quadruple wild-type GIST), targeted sequencing in two recent studies have shown the presence of activating mutations or gene fusions involving *FGFR1* [9,10]. In particular, one case harbored the fusion *FGFR1*–Hook homolog 3 (*HOOK3*) (in-frame fusion of *FGFR1* intron 17 and *HOOK3* intron-4) and two patients carried *FGFR1*–Transforming acidic coiled-coil-containing protein 1 (*TACC1*) transcripts (in-frame fusion of *FGFR1* intron 17 and *TACC1* intron-6) [9]. In these two fusions, *FGFR1* acts as the 5′ fusion gene and the breakpoint usually occurs in exons 17, 18, or 19. The FGFR1 extracellular, transmembrane, and kinase domains therefore remain intact (Figure 1A) [20]. These fusions have not been directly confirmed to be oncogenic in GIST, however, they have been reported in other cancers. In particular, a fusion between the *FGFR1* and *HOOK3* genes was reported in the case of acute lymphoblastic leukemia [21], whereas *FGFR1-TACC1* fusion is common in primary brain tumors such as extra-ventricular neurocytoma [22] and glioblastoma [23], and has been recently reported in a case of uterine spindle cell sarcoma [24].

The two activating missense mutations identified in FGFR1 were p.K656E [9] and p.N546K [10] (Figure 1C). Combining these two studies, 5/38 (10.5%) quadruple wild-type GIST had *FGFR1* alterations. Additionally, in another series of quadruple wild-type GIST, focal duplication of band 11q13.3 (involving *FGF3*/*FGF4*) was identified in 6/8 patients and this event was associated with the overexpression of FGF4, one of the most important ligands of FGFR1 [11] (Figure 1B). Altogether, these findings support the hypothesis of a potential involvement of FGFR pathway deregulation as a driver event in quadruple wild-type GIST.

The role of the FGFR pathway in GIST extends beyond quadruple wild-type GIST. In *SDHB*-deficient GIST, global DNA hyper-methylation is observed [25]. The methylation of specific insulator sequences prevents the binding of the transcriptional repressor CCCTC-binding factor (CTCF). This alteration ultimately leads to the overexpression of several potential oncogenes including FGF3 and FGF4 [26].

In c-KIT mutated GIST, the TKI imatinib represents the standard first-line therapy for patients with advanced disease and is also used in selected cases in the neoadjuvant and adjuvant settings [27]. Importantly, the FGFR signaling pathway has also been involved in the mechanisms of resistance to imatinib. In particular, FGF2 is overexpressed in imatinib-resistant GIST cells as well as in tumor samples from imatinib-resistant GIST [28,29]. Moreover, in complex crosstalk between tyrosine kinases, the interaction of FGF2 with FGFR1 and FGFR3, respectively, restored MAPK signaling during treatment with imatinib [29] and c-KIT phosphorylation in imatinib-resistant models [28]. A gain in *FGFR2* has also been suggested as an additional potential mechanism associated with imatinib resistance [30].

### 2.2. Rhabdomyosarcoma

RMS likely originates from a mesenchymal or skeletal muscle stem cell and is the most common STS in childhood. RMS is classified in four major histological subtypes: alveolar RMS (ARMS), embryonal RMS (ERMS), pleomorphic RMS, and spindle cell/sclerosing RMS. The different subtypes are characterized by different histological characteristics, molecular driver events, and prognosis [1]. Several different mechanisms of FGFR pathway alterations have been reported in RMS.

The first study that described aberrations in FGFR4 identified activating tyrosine kinase domain mutations in 7/94 (7.5%) of primary human RMS tumors [12]. In RMS cell line models, two of these mutations were shown to promote FGFR4 autophosphorylation, signal transducer and activator of transcription 3 (STAT3) phosphorylation, and activation of cell cycle and DNA replication pathways. Consequentially, FGFR4 mutations increased proliferation, invasion, and metastatic potential. Importantly, high expression of FGFR4 mRNA was also associated with worse survival in a clinical cohort of 146 patients [12]. In two separate cohorts, FGFR4 mutations were identified specifically in the ERMS subtype, where they showed a prevalence of ~7–8% [13,14]. Notably, they are often associated with genomic amplification of the mutant FGFR4 allele [31].

FGFR4 overexpression (Figure 1D) has also been found as a frequent event in ARMS cell lines (3/3 tested) as well as in tumor samples, with 34/39 (87.2%) samples showing an immunohistochemical signal in more than 25% of the cells [32]. This is mostly due to transcriptional activation by the oncogenic Paired box gene (*PAX*)3-Forkhead box protein O1 (*FOXO1*) and *PAX7-FOXO1* translocations [33,34]. In ARMS cells, FGFR4 stimulation induces degradation of the pro-apoptotic molecule Bcl-2-like protein 11 (BIM) and upregulation of its antagonist B-cell lymphoma-extra-large (Bcl-XL) [35]. The relevance of the FGFR pathway in the pathogenesis of ARMS is further confirmed by the identification of a *FOXO1-FGFR1* fusion gene as molecular driver in a case of ARMS [36]. Finally, in another study, RMS cell lines have been shown to harbor minor populations of FGFR3-positive cells characterized by stem cell properties with upregulation of undifferentiated cell markers and downregulation of differentiation markers and a cancer-initiating phenotype in vivo [37]. 

### 2.3. Liposarcomas

Liposarcomas are thought to arise from the malignant transformation of adipocytic stem cells. Based on the histological and molecular characteristics, three main subtypes of malignant liposarcoma are recognized: de-differentiated liposarcoma (DDLPS), characterized by the presence of the Mouse double minute 2 homolog (MDM2) gene amplification; myxoid/round cell liposarcoma, defined by the presence of a t(12;16) translocation resulting in the chimeric Fused in Sarcoma/Translocated in Liposarcoma (FUS)—DNA Damage Inducible Transcript 3 (DDIT3) fusion protein; and pleomorphic liposarcoma, a high-grade sarcoma characterized by the presence of pleomorphic lipoblast without specific molecular alteration [1,38]. 

However, rare events, two cases of DDLPS with a mutation in *FGFR1* and *FGFR3*, respectively, have been reported, and were characterized by a poor prognosis [39]. Besides activating mutations, the FGFR pathway can be activated via overexpression of its components. This has been reported for FGFR1 and FGFR4 in about 30% of DDLPS. Similarly to what was reported for the activating mutations, overexpression of FGFRs also correlated with a poor prognosis [40]. Moreover, the gene encoding for the FGFR adaptor protein *FRS2* is co-amplified with *MDM2* on chromosome 12q13–15 in about 90% of DDLPS (Figure 1E). The amplification is associated with overexpression of the protein and suggests that FGFR/FRS2 signaling may play a functional role in the development of high-grade DDLPS [15,16,17].

Among the other liposarcoma subtypes, overexpression of FGFR2 has been reported in myxoid liposarcoma both in primary tumor samples and cell lines. In the cell lines, FGFR2 regulates proliferation, apoptosis, and migration [41].

### 2.4. Phosphaturic Mesenchymal Tumor (PMT)

PMT is an exceptionally rare distinctive mesenchymal neoplasm displaying in most cases a benign behavior, although malignant cases have been described [42]. In about 60% of the cases, it is characterized from a molecular perspective by the presence of a Fibronectin 1 (*FN1)*-*FGFR1* fusion [43]. This event places the *FGFR1* gene under the constitutively active fibronectin promoter and ultimately leads to FGFR1 overexpression. FGFR1 transcriptionally upregulates its ligand *FGF23*. Notably, the FN1-FGFR1 chimeric protein is predicted to preserve its ligand-binding domains, thus effectively establishing an autocrine tumorigenic stimulus [43]. The secreted FGF23 can have endocrine effects on the kidney, leading to the loss of phosphate in the urine and the clinical syndrome of tumor-induced osteomalacia (TIO) [42]. In a minority of cases, the *FN1-FGF1* fusion gene has also been identified in PMT [44]. In these cases, the FN1-FGF1 protein is thought to be secreted and serve as a ligand that binds and activates FGFR1 to achieve an autocrine loop [44].

### 2.5. Malignant Rhabdoid Tumors

Malignant rhabdoid tumors (MRT), also known as extra-renal rhabdoid tumors or rhabdoid tumors of the soft-tissues, are aggressive pediatric cancers characterized by loss of the tumor suppressor SWI/SNF-related matrix-associated actin-dependent regulator of chromatin subfamily B member 1 (*SMARCB1*) [1]. In MRT cell lines and primary tumors, loss of *SMARCB1* was associated with increased expression of FGFRs, and this was paralleled in vitro by constitutive FGFR pathway activation and FGFR-dependency for growth [45]. In particular, through molecular profiling and chemical screens in MRT cell lines, FGFR1 and FGFR2 were independently confirmed to be key oncogenic drivers coactivated with PDGFRs [46,47].

### 2.6. Other Sarcomas

The FGFR pathway has been implicated in the development and progression of several other STS subtypes. Recently, Chudasama et al. described copy number gain and amplification of *FGFR1* analyzed by fluorescence in situ hybridization (FISH) respectively in 61/190 (32.1%) and 13/190 (6.8%) cases of high-grade sarcomas of various histologies [18]. Using array-based comparative genomic hybridization in a second cohort, they found *FGFR1* to be gained or amplified in 13/79 (16.5%) cases. These results were further confirmed using the Cancer Genome Atlas (TCGA) cohort of 254 sarcoma cases. Importantly, both the high-grade sarcoma and the TCGA cohorts showed that *FGFR1* mRNA overexpression could also occur in the absence of *FGFR1* copy number alterations [18]. This comprehensive analysis ultimately revealed FGFR1 copy number gain and overexpression in UPS, LMS, and DDLPS as well as other sarcoma subtypes [18]. In cellular models of STS from various histologies, the MAPK signaling axis was shown to be the most critical effector pathway mediating FGFR1 signaling [18]. Similarly, FGFR1 was also shown to be overexpressed in Ewing sarcoma, a high-grade mesenchymal malignancy of bone or soft tissue [48]. Additionally, Toulmonde et al. recently used a multi-omics platform to characterize UPS and identified a subgroup of UPS characterized by low expression of markers of immune activation. This subgroup showed an enrichment in genes involved in development and stemness including in particular *FGFR2*, which they propose to potentially represent a therapeutic target [49]. Similarly, *FGFR1* and *FGFR2* transcripts are enriched in subtype I of LMS, a transcriptionally defined molecular subtype characterized by the overexpression of genes involved in muscle contraction, muscle system processes, and cytoskeleton organization [50]. Finally, the expression levels of 22 *FGF* and four *FGFR* genes were analyzed in 18 primary tumors and five cell lines of synovial sarcoma. FGF ligands, in particular FGF8, were shown to have an important role in regulating cancer growth in vitro and in vivo through the MAPK signaling axis [51].

## 3. FGFR Inhibition in the Therapy of Gist and STS

### 3.1. Preclinical Evidence

Given its crucial role in the development and progression of several sarcoma subtypes, the FGFR pathway has emerged as a novel potential therapeutic target. Three main approaches are being evaluated: (1) the direct inhibition of the FGFR pathway via small molecule TKIs; (2) the indirect inhibition of the FGFR pathway through transcriptional modulation or inhibition of molecules involved in its signal transduction; and (3) the use of FGFR-antigen binding proteins such as aptamers or antibodies that could be used as stand-alone drugs or adapted as part of antibody–drug conjugates or chimeric antigen receptor (CAR) T cells [52].

Soon after the identification of activating mutations as well as overexpression of FGFR4 in RMS, FGFR TKIs were tested in vitro in RMS cell lines with promising activity [32]. Among these TKIs, ponatinib is one of the most potent FGFR4 inhibitors. Mechanistically, ponatinib suppressed phosphorylation of FGFR4 as well as its downstream target STAT3, leading to apoptosis in RMS cells. Treatment with this TKI also inhibited tumor growth in murine RMS xenograft models from cell lines expressing mutant FGFR4 [53]. 

The use of FGFR TKIs has also been studied in preclinical models of several other sarcoma subtypes beside RMS. In particular, FGFR inhibition with the pan-FGFR inhibitor TKIs infigratinib and LY2874455 has shown preclinical activity in *FRS2*-amplified DDLPS cell lines, with evidence of strong inhibition of cell proliferation and accumulation of cells in the G0 phase of the cell cycle [54,55]. It has, however, been suggested that *FRS2* amplification alone might not be sufficient to predict response, and that the expression of FGFR ligands such as FGF2 may also contribute to the activity of FGFR inhibitors in DDLPS [55]. In FGFR2-overexpressing myxoid liposarcoma cell lines, the FGFR TKIs PD173074, dovitinib and infigratinib, reduced cell proliferation and migration, and this effect was further increased by their combination with the standard chemotherapeutic agent trabectedin [41]. Recently, the FGFR TKIs erdafitinib, infigratinib, and AZD4547 have been shown to decrease in vitro viability, FGFR2 phosphorylation, and downstream signaling, and induced G0–G1 arrest in cells derived from immune-low UPS, which are characterized by the overexpression of FGFR2 [49]. Finally, pharmacological FGFR inhibition significantly impaired the growth of patient-derived xenografts from immune-low UPS, but had no effect on those from immune-high UPS [49]. Similarly, the TKIs ponatinib, dovitinib, infigratinib, and PD173074 have been tested in preclinical models of Ewing sarcoma, where they have shown the capability to suppress cellular proliferation [56,57]. Direct inhibition of FGFR with infigratinib has also been investigated in preclinical models of SDH-deficient GIST, where it resulted in complete suppression of tumor growth in vivo throughout the dosing period [26]. In imatinib-resistant GIST models, simultaneous treatment with infigratinib and imatinib was associated with reduced tumor proliferation in vitro and in vivo, where major histopathologic changes were also observed [29,58]. Moreover, in vitro evidence suggested that infigratinib induced sensitization of GIST to topoisomerase II inhibitors, leading to increased numbers of apoptotic cells in FGFR-inhibited GIST cells after treatment with doxorubicin [59,60].

Besides via direct inhibition of FGFR receptors with TKIs, the FGFR pathway can also be inhibited indirectly. In models of RMS, treatment with guadecitabine, a next-generation DNA methyltransferase inhibitor that significantly alters the epigenetic and transcriptional landscape, was associated with the downregulation of FGFR4 mRNA and protein levels and inhibited cellular proliferation [61]. In a murine model of RMS induced by orthotopic injection of myoblasts transduced with *FGFR4*-activating mutations N535K and V550E, PI3K inhibitors were identified in a drug screen as the most potent inhibitors of cell proliferation. Omipalisib, a dual PI3K/mechanistic target of rapamycin (mTOR) inhibitor, selectively inhibited the growth of murine and human RMS cells carrying activating mutations in *FGFR4* [62]. Indirect inhibition of the FGFR pathway can also be achieved by binding and sequestering of FGFs. This is one of the physiological roles of the soluble pattern recognition receptor long pentraxin-3 (PTX3) [63] and is being investigated as a therapeutic strategy with the development of extracellular “FGF ligand traps” [64]. In a fibrosarcoma model, PTX3 overexpression or treatment with the small molecule pan-FGF trap NSC12 significantly reduced the proliferative and tumorigenic potential of fibrosarcoma cells in vitro and in vivo [65].

Finally, FGFRs can also be directly targeted by binding antibodies. Recently, single-domain antibodies selectively binding to FGFR4 have been identified. In vitro, preincubation of FGFR4 expressing cells with these antibodies resulted in the abolishment of ligand-induced MAPK phosphorylation. Once immobilized on the surface of vincristine-loaded liposomes, these single-domain antibodies were able to be recognized by FGFR4 expressing RMS cells and internalized. Moreover, one of these antibodies was used to generate CAR T cells targeting FGFR4 and was shown to mediate significant antitumor activity against FGFR4-expressing RMS cells in vitro [66].

### 3.2. Clinical Evidence: Multi-Target TKIs 

There are a number of broad spectrum multi-target TKIs that inhibit a number of different kinases including the FGFR family. These include pazopanib, brivanib, S49076, nintedanib, lenvatinib, dovitinib, and ponatinib. Among the multi-target TKIs inhibiting FGFRs, pazopanib has obtained regulatory approval by the Food and Drug Administration (FDA) and European Medicines Agency (EMA) for the treatment of patients with non-adipocytic STS after the failure of standard chemotherapy [67]. Pazopanib is a multi-target TKI with activity against vascular endothelial growth factor receptors (VEGFRs), PDGFRs, c-KIT, FGFRs, and Colony-stimulating factor-1 receptor (CSF1R) amongst other tyrosine kinases [68]. Pazopanib has shown activity in patients affected by advanced non-adipocytic STS in a randomized placebo-control phase III clinical trial [69]. In particular, treatment with pazopanib was associated with a significant increase in progression-free survival (PFS) (4.6 months vs. 1.6 months in the placebo arm) and a non-significant increase in overall survival (12.5 months vs. 10.7 months in the placebo arm) [69]. Pazopanib has also been prospectively investigated in specific rare sarcoma subtypes, which are believed to be sensitive to its activity such as solitary fibrous tumor and extraskeletal myxoid chondrosarcoma [70,71,72]. 

A number of other multi-target TKIs with activity against FGFRs beyond pazopanib have been and are currently being investigated in STS [73]. Brivanib is a selective inhibitor of VEGFRs and FGFRs, whose activity in STS patients has been investigated within a multi-cohort, phase II, randomized, discontinuation trial. The trial enrolled a total of 595 patients including 251 STS patients. Radiological responses were seen in seven patients, three affected by angiosarcoma. For all randomized patients with STS, the median PFS was 2.8 months for those treated with brivanib compared with 1.4 months for the placebo. This trial showed that FGF2 expression is not a predictive biomarker of the efficacy of brivanib [74]. S49076 is a novel ATP-competitive TKI of MET, AXL, and FGFRs, whose safety has been tested in a phase I trial. In this study, one patient with synovial sarcoma was reported to have a stable disease lasting at least six months [75]. Nintedanib is active against VEGFRs, PDGFRs, and FGFRs [76] and its activity has been explored in a phase II trial randomizing advanced unselected STS patients to receive ifosfamide or nintedanib as second-line therapy. This trial was recently stopped for futility [77].

There are also a number of ongoing trials. Anlotinib is a recently developed multi-target TKI targeting a similar spectrum of targets [78], and it is currently being evaluated in patients affected by alveolar soft part sarcoma, LMS, and synovial sarcoma (NCT03016819). Similarly, lenvatinib is a potent inhibitor of VEGFs, FGFRs, PDGFRα, c-KIT, and rearranged during transfection (RET) [79]. Based on synergies observed in other tumor types, its activity is being evaluated in sarcomas in combination with the inhibitor of microtubule dynamics eribulin (NCT03526679) and with the checkpoint inhibitor pembrolizumab (NCT04784247).

In GIST, multi-target TKIs with activity against FGFRs have also been tested. In particular, dovitinib targets FGFRs, PDGFRs, VEGFRs, and c-KIT [80] and was tested as a second-line treatment in patients refractory to imatinib or who do not tolerate imatinib, and showed signs of activity, with a disease control rate at 12 weeks of more than 50% [81]. Similarly, the multi-target TKI ponatinib is also being investigated in patients with metastatic and/or unresectable GIST after failure of prior TKI therapy (NCT01874665). Preliminary results showed that ponatinib has clinical activity in advanced GIST patients after TKI failure, particularly those with *KIT* exon 11 mutations [82].

### 3.3. Clinical Evidence: Selective FGFR TKIs

In the last few decades, TKIs selectively targeting FGFRs have been developed and are currently under investigation in several different cancer types. In particular, through the FDA accelerated approval process, erdafitinib is indicated to treat metastatic urothelial carcinoma with FGFR2 and FGFR3 alterations, and pemigatinib is indicated to treat unresectable cholangiocarcinoma with fusions or rearrangements of FGFR2 [83]. Recently, the EMA’s Committee for Medicinal Products for Human Use has also adopted a positive opinion, recommending the granting of a conditional marketing authorization for pemigatinib in cholangiocarcinoma [84]. Although preclinical evidence supporting the use of selective FGFR inhibitors in sarcomas is constantly increasing, their clinical development in the treatment of STS patients is still at an early stage. This is further complicated by the rarity of individual sarcoma subtypes, which hampers the design of subtype specific clinical trials.

Selective FGFR TKIs can be categorized based on the number and types of FGFRs they inhibit, on the reversibility of their inhibition, and on their mechanism of action [85]. TKIs tested or currently under evaluation in sarcomas include the FGFR1-3 inhibitors Debio1347 and infigratinib, and the FGFR1-4 inhibitors futibatinib, rogaratinib, and ASP5878 (Table 2). Several of these TKIs have, however, been tested only in early phase clinical trials and therefore there are no data specifically for sarcoma patients, although some have been enrolled in these trials [52]. It is not possible to accurately estimate the number of STS patients treated with FGFR inhibitors thus far [86,87].

Among the available information, one sarcoma patient was included in the phase I study of the selective FGFR1-3 inhibitor Debio1347 in patients with tumors harboring FGFR gene alterations (NCT01948297). In this patient, the best response was progressive disease [88]. Two sarcoma patients were enrolled in the phase I study of the irreversible FGFR1-4 inhibitor futibatinib (NCT02052778). The best responses were stable disease for one patient and progressive disease for the other [89]. One patient affected by LMS was enrolled in the phase I study of ASP5878 (NCT02038673), a FGFR1-4 TKI. No information on radiological response was reported [90]. 

Among the trials currently enrolling, one is selectively enrolling STS and GIST patients: in this trial, rogaratinib, an FGFR1-4 TKI, will be tested in patients with sarcomas harboring alterations in FGFR1-4 identified by next-generation sequencing profiling, and in patients with advanced SDH-deficient GIST (NCT04595747).

With regard to FGFR inhibitors specifically used in sarcomas, there is a recent case report of a patient with TIO associated with metastatic PMT in which treatment with infigratinib, a FGFR1-3 TKI, dramatically decreased bone lesions [92]. A phase II trial testing infigratinib in TIO has recently closed, and the full results are awaited (NCT03510455). Moreover, in patients with imatinib refractory advanced GIST, a phase Ib trial evaluated the safety and efficacy of infigratinib in combination with imatinib. In this population, stable disease ≥32 weeks was observed in three of 12 evaluable patients [91].

### 3.4. Clinical Evidence: Non-TKI FGFR-Specific Inhibitors

FP-1039, a FGF ligand trap, was tested in a phase I in patients with advanced solid tumors including six patients affected by sarcomas. No responses were observed in this study and there was no apparent relationship between best tumor response and the presence of FGF2 and FGFR1 protein overexpression or *FGFR1* gene amplification [93]. Finally, one patient affected by GIST was enrolled in the first-in-human trial of aprutumab ixadotin, a FGFR2 antibody-drug conjugate (NCT02368951), with progressive disease as the best response [94].

## 4. Conclusions

Since the first report of the activity of imatinib in a GIST patient [95], the development of targeted therapies has radically changed the treatment paradigm in several cancer types. This has been made possible by the increasing integration of gene sequencing into clinical practice [96]. The identification of alterations in the FGFR pathway in cancer has accelerated the development of drugs targeting this signaling axis. In STS, the multi-target TKI pazopanib has been approved by the FDA and EMA and several other related drugs are currently under investigation. It is, however, difficult to infer in these cases the relative contribution of FGFR inhibition compared to the inhibition of other phylogenetically related oncogenic tyrosine kinase receptors such as VEGFRs and PDGFRs. In fact, there is general uncertainty over whether these multi-target TKIs sufficiently inhibit FGFRs in the clinical setting as dosing is usually limited by hypertension as a result of VEGFR inhibition and by non-specific toxicity [97].

The evidence for the clinical impact of FGFR inhibition in STS patients is currently very limited and for the most part still under investigation. It is also important to consider that response rates seen with FGFR inhibitor therapy in multiple cancer histologies (around 20–40%) are significantly below those observed for patients with other oncogenic fusions such as those involving *ALK*, *ROS1*, and *NTRK* (around 60–70%) [98]. Moreover, with the exception of cases of PMT and associated TIO, there have been virtually no reports of STS patients treated with selective FGFR inhibitors with sustained tumor response. Additional data from phase II studies specifically testing FGFR inhibitors in STS and GIST are required to better characterize the potential role of FGFR inhibition as a monotherapy in STS.

Given the limited activity of selective FGFR TKIs in STS, it will become particularly important to understand the molecular alterations associated with signs of activity. This could lead to the identification of molecular biomarkers that could be used for patient stratification. An example of such an effort is the Phase 2 randomized discontinuation trial of brivanib, which showed that FGF2 expression (as defined in the trial protocol) was not a marker of the efficacy of brivanib [74]. Importantly, beyond TKIs, novel strategies are being developed to selectively inhibit the FGFR pathway including FGF traps and FGFR antibodies. The latter in particular, besides directly inhibiting the receptors, might take advantage of FGFR overexpression on cancer cells to promote engagement by immune cells or more selectively deliver chemotherapy.

Selective FGFR inhibition might prove clinically ineffective as a monotherapy in STS patients despite promising preclinical evidence due to several reasons: FGFRs might be amplified only in a subpopulation of cells, therefore representing a non-driver event; variable addiction to FGFR amplification might be modulated by the expression or mutation of other transducing molecules such as components of the MAPK pathway; and activating alternative oncogenic pathways (in particular VEGFR and PDGFR) that may compensate for FGFR inhibition [97]. Ongoing and future preclinical investigations and clinical trials will ultimately shed light on whether FGFR inhibition in STS might represent a valid clinical target in a selected subpopulation of patients.

## Figures and Tables

**Table 1:** Estimated frequency of FGFR pathway alterations in GIST and STS.

Disease	Alterations	Estimated Frequency	Ref.
GIST	Translocation, duplication, activating mutations	~1–2%	[9,10,11]
Rhabdomyosarcoma	Activating mutations	~7–8%	[12,13,14]
Dedifferentiated liposarcoma	Amplification, activating mutations	>90%	[15,16,17,18]
Undifferentiated pleomorphic sarcoma	Amplification	8–16%	[18]
Leiomyosarcoma	Amplification	~30%	[18]
Malignant peripheral nerve sheath tumors	Amplification	11–20%	[18]

**Table 2:** Clinical trials evaluating selective FGFR TKIs in sarcoma patients.

Drug	FGFR Selectivity	Phase	Sarcoma Subtypes Enrolled	Best Response of Sarcoma pts.	National Clinical Trial (NCT) Number	Ref.
Erdafinitib	1–4	I	Any subtype	N/A	NCT01703481	[86]
Debio1347	1–3	I	Any subtype harboring FGFR gene alterations	1 PD	NCT01948297	[88]
Futibatinib	1–4	I	Any subtype harboring FGFR gene alterations	1 SD, 1 PD	NCT02052778	[89]
ASP5878	1–4	I	Any subtype	N/A	NCT02038673	[90]
Infigratinib + imatinib	1–3	Ib	Imatinib refractory advanced GIST	3/12 SD	NCT02257541	[91]
Infigratinib	1–3	II	Any subtype associated to TIO	N/A	NCT03510455	
Rogaratinib	1–4	II	Any subtype with FGFR1-4 gene alterations + advanced SDH-deficient GIST	Currently enrolling	NCT04595747	
